# B Cell Responses against Influenza Viruses: Short-Lived Humoral Immunity against a Life-Long Threat

**DOI:** 10.3390/v13060965

**Published:** 2021-05-22

**Authors:** Jenna J. Guthmiller, Henry A. Utset, Patrick C. Wilson

**Affiliations:** 1 Section of Rheumatology, Department of Medicine, University of Chicago, Chicago, IL 60637, USA; hutset@medicine.bsd.uchicago.edu (H.A.U.); wilsonp@uchicago.edu (P.C.W.); 2 Committee on Immunology, University of Chicago, Chicago, IL 60637, USA

**Keywords:** influenza viruses, humoral immunity, broadly neutralizing antibodies, imprinting, hemagglutinin, germinal center, plasma cells

## Abstract

Antibodies are critical for providing protection against influenza virus infections. However, protective humoral immunity against influenza viruses is limited by the antigenic drift and shift of the major surface glycoproteins, hemagglutinin and neuraminidase. Importantly, people are exposed to influenza viruses throughout their life and tend to reuse memory B cells from prior exposure to generate antibodies against new variants. Despite this, people tend to recall memory B cells against constantly evolving variable epitopes or non-protective antigens, as opposed to recalling them against broadly neutralizing epitopes of hemagglutinin. In this review, we discuss the factors that impact the generation and recall of memory B cells against distinct viral antigens, as well as the immunological limitations preventing broadly neutralizing antibody responses. Lastly, we discuss how next-generation vaccine platforms can potentially overcome these obstacles to generate robust and long-lived protection against influenza A viruses.

## 1. Introduction—Current Pitfalls of Humoral Immunity against Influenza Viruses

Humans are exposed to influenza A viruses (IAVs) throughout their lifetime. As a result, virus-specific B cells are periodically stimulated to generate antibody responses against viral antigens that can help eliminate the virus, prevent viral spread, and provide protection against future infections. Antibodies against the surface glycoprotein hemagglutinin (HA) are a key correlate of protection against influenza viruses [1]. Therefore, current influenza virus vaccines largely function to elicit antibodies against HA to provide protection against IAV infections.

However, humoral immunity against IAVs is short-lived for multiple reasons, including virus- and host-specific factors. First, influenza viruses are antigenically variable. Numerous IAV subtypes exist, with humoral immunity against one subtype providing little, if any, protection against other subtypes, including IAVs of zoonotic origin. Moreover, individual IAV subtypes have numerous individual strains that have evolved over time to circumvent host immunity, with most antibodies providing protection against only a few strains of a given IAV subtype. Strain diversification is largely driven by the acquisition of antibody escape mutations within the major surface glycoproteins HA and neuraminidase (NA), a process referred to as antigenic drift. Moreover, antigenically novel IAV subtypes in humans can arise from spillovers of newly recombined IAV subtypes in animals, a process known as an antigenic shift. Notably, antigenically shifted viruses are responsible for four IAV pandemics in the last 103 years. Therefore, secreted antibodies and memory B cells (MBCs) generated by an exposure early on in life are unlikely to provide protection against infection against an antigenically advanced IAV strain of the same subtype or a novel subtype later in life. The lack of robust and broad protection from IAV subtypes and strains necessitates the yearly reformulation of seasonal influenza virus vaccines.

In addition to the highly variable nature of IAVs, antibodies against HA largely bind epitopes that are permissible to mutating, rather than broadly neutralizing epitopes, for unclear reasons [2,3]. People are more likely to recall MBCs from prior exposures to respond to a new IAV drifted variant, which can induce antibodies against poorly protective or non-protective internal antigens or against epitopes of HA and NA that are prone to mutating [4]. Moreover, serum antibodies against IAVs decline after viral exposure [5,6], further exacerbating the short-lived nature of humoral immunity against influenza viruses. In this review, we will discuss how humoral immunity against IAVs is generated and recalled by different viral exposures. Furthermore, we will highlight the recent advances in our understanding of why protective humoral immunity against IAVs is short-lived and how different vaccine strategies have the potential to overcome these obstacles to elicit broad and long-lived protection against IAVs.

## 2. Antibody Responses against Distinct Viral Antigens and Epitopes

### 2.1. Antibodies against HA

**Figure 1:**
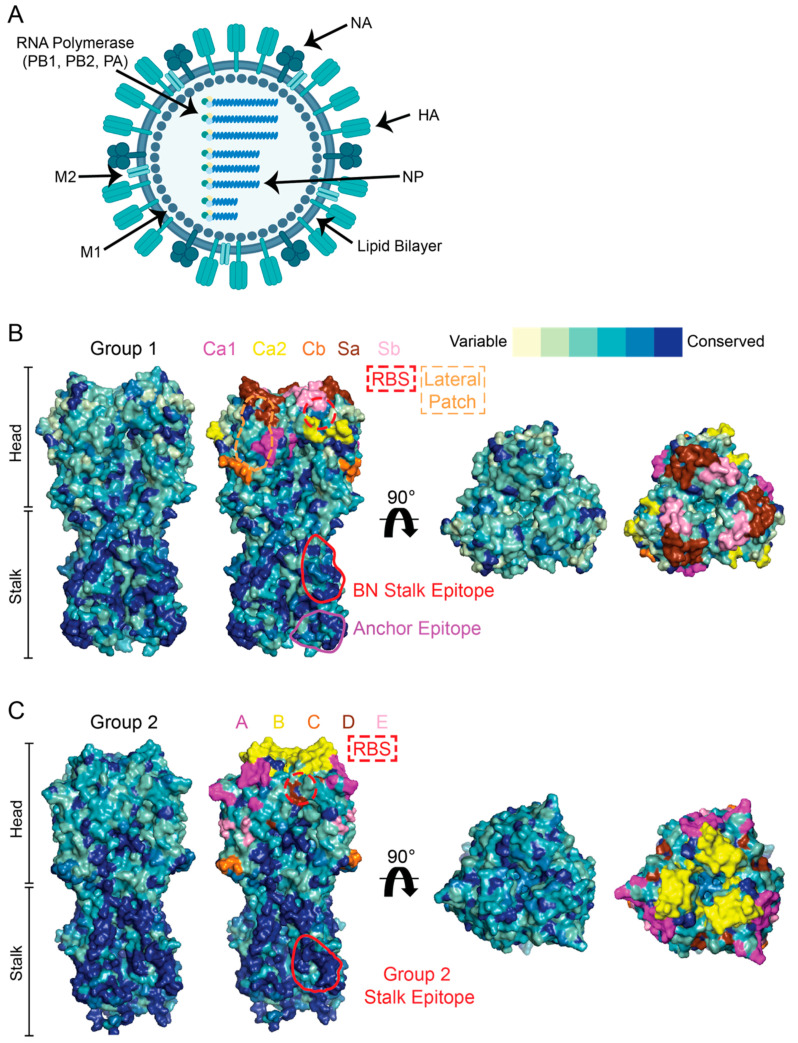
IAV structure and conservation of HA. (**A**) Illustrated structure of IAVs. (**B**) Conservation of group 1 HA with variable epitopes (Ca1, Ca2, Cb, Sa, Sb) and conserved epitopes (RBS, lateral patch, broadly neutralizing (BN) stalk epitope, and anchor epitope) highlighted. (**C**) Conservation of group 2 HA with variable epitopes (A, B, C, D, E) and conserved epitopes (RBS and group 2 stalk epitope) highlighted.

The genome of IAVs consists of eight negative-sense viral RNA segments, with each expressing one to two distinct proteins, leading to the potential expression of a total of 10 to 14 viral proteins (Figure 1A). HA is the most abundant surface protein and is responsible for binding to the host receptor, sialic acid, and mediating the infection of a host cell. In accordance, most antibodies induced by seasonal vaccination target HA to neutralize the virus and prevent it from infecting host cells [1,2,4]. HA is expressed as a single precursor protein (HA0) that is cleaved by host proteases into two subunits (HA1 and HA2). Moreover, the HA protein is divided into two functionally distinct domains: the head and the stalk (Figure 1B,C). The head domain is generated from the HA1 subunit and possesses the receptor-binding site (RBS) that mediates HA binding to sialic acid. The stalk domain, also commonly referred to as the HA stem, extends into the viral membrane and is responsible for fusing the viral and host membranes in a pH-dependent manner to allow for the endosomal release of the internal viral contents and productive infection. The HA stalk domain is primarily comprised of HA2. IAVs can be divided into 18 distinct HA subtypes (H1-18), making up two distinct phylogenetic groups: group 1 and group 2. Currently, H1- (group 1) and H3- (group 2) expressing viruses cause seasonal IAV infections in humans. Notably, the HA head domain is more variable compared to the stalk domain across the group 1 and group 2 viruses (Figure 1B,C).

Neutralizing antibodies against HA function to inhibit viral entry into a host cell by blocking receptor binding, inhibiting viral membrane fusion, or interfering with viral egress. Most antibodies against the HA head inhibit receptor binding, which is commonly determined using hemagglutination inhibition (HAI) assays. Most antibodies elicited by seasonal vaccination target variable HAI^+^ epitopes of the HA head and therefore have limited viral neutralization breadth [2,4]. Both group 1 and 2 IAVs each have five distinct major antigenic sites (Figure 1B,C), with humans exhibiting discrete immunodominance patterns towards these epitopes [7,8,9]. Moreover, the HA epitopes regularly targeted by HAI^+^ antibodies evolve more rapidly and are more able to escape variants than conserved regions of HA [3,10,11]. Therefore, head-specific antibodies drive the evolution of influenza viruses to become resistant to antibodies targeting particular head epitopes.

More rarely, antibodies are elicited against two broadly neutralizing HAI^+^ epitopes: the RBS and the lateral patch (Figure 1B) [12,13,14,15,16,17,18]. Antibodies against the RBS are potently neutralizing across viruses within a given subtype [15,17,18], and can occasionally cross-react with other subtypes [12,19]. However, cross-reactivity to other subtypes often comes at the cost of limited strain reactivity within a given subtype [12]. The broad strain and subtype reactivity of RBS-binding antibodies is likely driven by serial exposure to distinct IAV subtypes, as RBS-binding antibodies demonstrate a stronger affinity to historical HAs [19,20]. Antibodies against the lateral patch are broadly neutralizing against H1 viruses, but rarely cross-react with other subtypes [13,21]. Little is known about the affinity maturation of B cells against the lateral patch. Moreover, recent mutations in the major antigenic site Sa of H1N1 viruses have ablated antibody binding to the lateral patch, potentially due to antibody pressure against this epitope [13,16]. Despite this, nearly one-third of lateral patch binding antibodies maintain neutralization against recent H1N1 (pH1N1) pandemic viruses [20], indicating that the lateral patch is still an important epitope.

Not all antibodies against the HA head are HAI^+^, as antibodies against the vestigial esterase epitope on the side of the head domain do not possess HAI activity, but do mediate neutralization by inhibiting membrane fusion and possess antibody-dependent cellular cytotoxicity (ADCC) activity [22,23]. Similar to antibodies targeting the lateral patch, vestigial esterase-binding antibodies exhibit broad reactivity against strains within a particular subtype, but do not cross-react with other subtypes [22]. Moreover, antibodies against a newly identified head epitope at the trimer interface of HA are HAI^-^ and non-neutralizing, but do provide potent protection in in vivo mouse models via ADCC [24,25,26]. Trimer interface-binding antibodies exhibit broad viral breadth across IAV subtypes, with many antibodies binding seasonal IAV subtypes and zoonotic viruses [25,26]. Therefore, antibodies against distinct epitopes of the HA head domain can provide protection via multiple mechanisms, including by neutralizing receptor binding, inhibiting viral membrane fusion, and mediating ADCC.

The HA stalk domain is critical for pH-mediated fusion of the viral membrane with the host membrane. In accordance, antibodies binding the HA stalk frequently neutralize IAVs by inhibiting viral membrane fusion and by limiting cleavage of HA0 into the HA1 and HA2 subunits [27,28]. Moreover, antibodies against the HA stalk can prevent viral egress by inhibiting NA activity and cross-linking HA trimers across virions [28,29,30,31]. Importantly, neutralizing and non-neutralizing antibodies binding distinct epitopes of the HA stalk can provide in vivo protection via Fc receptor (FcR)-mediated functions, including ADCC [32,33,34]. Therefore, stalk-binding antibodies can provide protection through multiple distinct mechanisms.

The HA stalk domain is generally more conserved and less permissive to mutations than the HA head domain [3], although escape variants from broadly neutralizing antibodies (bnAbs) targeting stalk epitopes have been identified [35,36,37,38]. However, viral mutants generated to evade stalk-targeting bnAbs often have reduced pathogenicity [11,37]. Moreover, antibodies against the HA stalk are less likely to be induced relative to the HA head by seasonal influenza virus vaccines [2,4], indicating that the stalk domain is immunosubdominant. The high degree of conservation of the HA stalk domain is likely the result of the stalk being less permissive to mutations and the fact that fewer antibodies are generally elicited against it. Therefore, there is less immune pressure on the HA stalk domain and viruses that do mutate in this domain experience a more selective fitness bottleneck relative to IAVs that mutate in the HA head domain.

Similar to the HA head domain, the HA stalk domain has multiple conserved epitopes that are targeted by bnAbs. The first bnAbs against HA were identified against the broadly neutralizing stalk epitope (Figure 1B) [27,39,40]. Antibodies binding the broadly neutralizing stalk epitope frequently neutralize group 1 IAVs [27,41] and can occasionally cross-react and neutralize group 2 IAVs [37,39,42,43,44]. Notably, one antibody binding the broadly neutralizing stalk epitope, CR9114, can neutralize group 1 and 2 IAVs as well as provide protection against influenza B viruses [39]. A second class of bnAbs targeting a membrane-proximal anchor epitope of the H1 stalk (Figure 1B) were recently identified and are broadly neutralizing across H1-expressing viruses [45,46]. Lastly, a third class of bnAbs targeting the stalk domain of group 2 viruses (Figure 1C) have been characterized and have a distinct binding footprint relative to antibodies targeting the broadly neutralizing stalk epitope [47,48]. Importantly, antibodies against the HA stalk are an independent correlate of protection against IAV infection [1,49]. Therefore, vaccines that preferentially induce antibodies against the stalk can provide broad protection against IAV infection.

### 2.2. Antibodies against NA

NA is the other major surface glycoprotein of influenza viruses and is critical for viral egress by cleaving HA binding to sialic acids, thereby allowing for the release of virions. IAVs utilize 1 of 11 NA proteins that combines with the 1 of 18 HA proteins to form distinct IAV subtypes (e.g., H1N1, H3N2). Structurally, NA is a tetramer composed of four identical polypeptides and, similar to HA, NA is comprised of two domains: the head and stalk domains. Although NA can undergo antigenic drift, NA generally mutates discordantly and at a slower rate compared to HA [50,51,52]. Similar to HA proteins, NA proteins are divided into antigenic clusters: group 1 (e.g., N1) and group 2 (e.g., N2). Notably, N10 and N11 are bat NA-like proteins that do not fall into either antigenic group and are specifically expressed with the bat-associated HA proteins H17 and H18 (group 1 HAs).

NA-reactive antibodies largely target the globular head domain and inhibit the enzymatic properties of NA, preventing the release of nascent virions [53]. Antibodies inhibiting NA activity are characterized as NAI^+^ and pre-existing NAI^+^ serum responses are associated with protection from IAV infection and disease [54,55]. Most characterized mAbs against the NA head have been found to directly bind or bind near the active site [56,57], which are frequently broadly reactive within NA subtypes [58,59], NA groups [57], and sometimes across NA groups of IAVs or NAs from influenza B viruses [56,60]. Therefore, NA-reactive antibodies can prevent infection against nearly all strains within a particular IAV subtype and potentially across NA groups. Moreover, NA-reactive antibodies have the potential to be administered as therapeutics to limit influenza disease, particularly against IAVs that are resistant against the licensed antiviral oseltamivir [51]. Whether antibodies can be induced against the NA stalk is not well understood. It has been proposed that the mushroom-like shape of NA head sterically blocks B cells from binding epitopes on the NA stalk, accounting for our limited knowledge of the NA stalk and immunity against it.

NA-binding antibodies are poorly induced by vaccination and have largely only been detected following IAV infections [4,56,57,61,62]. Notably, NA antigenicity is reduced during vaccine preparation and manufacturing [57], limiting the ability of MBCs to be recalled against NA by vaccination. Moreover, the quantity of NA included in current seasonal influenza virus vaccines is not standardized and NAI^+^ serum titers are rarely assessed [63]. Therefore, more research is needed to understand how to improve the antigenicity of NA within influenza virus vaccines.

### 2.3. Antibodies against M2 and Internal Antigens

Matrix protein 2 (M2) is an ion channel located within the viral membrane. (Figure 1A). The N-terminus of the M2 ectodomain (M2e) is highly conserved across IAVs [64]. However, antibodies are not commonly induced against M2 [65,66], which is likely because M2e is overshadowed by the larger and densely packed HA and NA proteins on the viral surface. Although anti-M2 antibodies are non-neutralizing, anti-M2 antibodies have the potential to provide broad protection against IAVs via ADCC and potentially complement-dependent cellular cytotoxicity (CDCC) [67,68,69,70]. Moreover, human anti-M2e mAbs can provide protection against lethal IAV infections in mice [64]. Whether anti-M2e antibodies contribute to protective humoral immunity in humans is not known.

Nucleoprotein (NP) is an abundant highly conserved internal protein expressed by IAVs and functions to encapsulate the segmented ssRNA genome (Figure 1A). Many reports have shown that antibodies against NP are induced by natural infection and vaccination [47,71,72] and are broadly reactive [4,51]. Whether antibodies against NP can protect against infection is controversial, as anti-NP antibodies are non-neutralizing [4]. NP immunization in mice has been shown to provide protection against IAV infection in an antibody-dependent manner [73,74]. Moreover, anti-NP antibodies correlate with serum ADCC antibody titers [75]. However, antibodies against NP are not a correlate of protection against IAV infection in mice [76]. Moreover, passive transfer of human mAbs against NP provide minimal protection against lethal IAV infection in mice [4]. Therefore, prospective studies are needed to understand whether anti-NP antibody titers correlate with protection against infection.

Antibodies can also be generated against other internal proteins, including matrix protein 1 (M1), the viral polymerases (PB1, PB2, and PA), nuclear export protein (NEP), and non-structural protein 1 (NS1). However, the general seroprevalence of antibodies against these epitopes is not well defined, although antibodies against these antigens have been observed [77,78,79,80,81,82]. Notably, M1, the inner scaffolding protein, is immunogenic as serum antibodies against M1 were detected following IAV infection [75,83,84]. The potential role of antibodies targeting these internal antigens is also unknown, as antibodies targeting internal proteins are typically non-neutralizing. The general quantity of these internal proteins, with the exception of M1, is considerably lower than that of HA, NA, and NP and, therefore, antibodies may be rarely induced against these antigens [85]. As these antigens are highly conserved across IAVs, antibodies against internal viral antigens are likely broadly reactive. However, the potential of antibodies against these antigens to provide protection against IAV infections remains unknown.

## 3. Generation and Recall of Humoral Immunity against IAVs

### 3.1. Humoral Immunity upon First Exposure to IAVs

**Figure 2:**
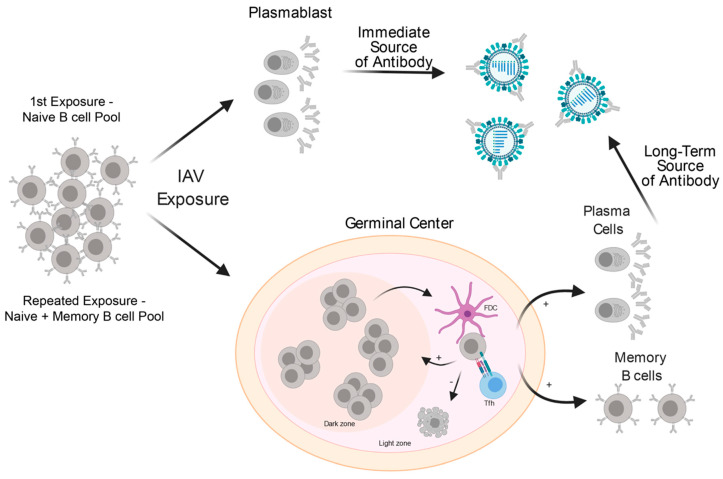
B cell activation trajectories after IAV exposure. Upon IAV exposure, naïve B cells (1st exposure and repeated exposures) and memory B cells (repeated exposures only) can differentiate into antibody-secreting plasmablasts or enter into the germinal center to affinity mature against IAV antigens and differentiate into long-lived plasma cells or memory B cells.

Upon first exposure to an influenza virus, naïve B cells specific to discrete viral antigens become activated and can become extrafollicular plasmablasts (PBs) or germinal center (GC) B cells (Figure 2). Most naïve B cells specific to protein antigens become activated in a T-dependent manner, requiring CD4 T cell assistance to differentiate into PBs or GC B cells [86]. PBs derived from naïve B cells are a short-lived antibody-secreting cell type that largely produce low affinity, albeit high avidity IgM [87]. PBs generated upon first exposure are believed to provide an immediate source of protective antibodies, while affinity maturation and clonal selection take place within the GC. However, the precise role and function of naïve B cell-derived PBs in humans are not known.

Within days of IAV exposure, GCs begin to form with two defined anatomical regions: the dark zone and the light zone (Figure 2). Within the dark zone, B cell clones against distinct viral epitopes proliferate and mutate their respective antibody genes, which comprise the antigen-binding sites of the B cell receptor (BCR), with the goal of increasing affinity for their specific epitope. Successful B cell clones mutate their BCRs to increase intraclonal diversity without causing deleterious mutations, such as those that would result in the BCR genes being out of frame and unable to be expressed [88,89]. If successfully mutated without causing deleterious mutations, GC B cells can travel from the dark zone to the light zone to compete for and internalize antigen presented by follicular dendritic cells. B cell clones that have captured and internalized antigen will present antigen peptides via MHC-II to T follicular helper (Tfh) cells with the same antigen-specificity. Notably, the highest-affinity clones are capable of capturing antigen and in turn, present antigen to Tfh cells that stimulate and provide pro-survival signals to GC B cells [90]. Moreover, selected GC B cells undergo more rounds of proliferation in a Myc dependent fashion [91,92]. B cells that do not successfully capture antigen and present it to Tfh cells eventually undergo apoptosis due to neglect [93]. GC B cells that receive Tfh help can either re-enter into the dark zone to undergo further affinity maturation or differentiate into either long-lived plasma cells (LLPCs) or MBCs. Accumulating evidence suggests that the highest affinity B cells are selected for entry into the LLPC pool, whereas lower affinity B cells are more likely to differentiate into MBCs [94,95,96,97]. In the context of flaviviruses, the LLPC pool tends to be high affinity but generally more strain specific, whereas the MBC pool is more flexible to viral variants [94,95]. It remains to be determined whether IAV-specific LLPCs and MBCs demonstrate similar affinity-based bifurcation of B cells. Notably, HA-specific MBCs have been shown to have a range of affinities, including those with low or undetectable affinities [19,98], whereas MBC-derived PBs have a high affinity [40,99]. The potential role of MBC clones with low and undetectable affinities in the generation of protective humoral immunity remains to be explored.

In theory, upon first exposure to an IAV subtype, B cells against all IAV specificities can become activated and participate in the humoral immune response. However, several factors limit which specificities are recruited into the humoral immune response, including the quantity and affinity of the naïve B cell repertoire against a particular antigen, the relative abundance of distinct viral antigens, the accessibility of particular epitopes on a given antigen, and the repertoire and quantity of CD4 T cells that can complement antigen-specific B cells. Moreover, the exposure route plays a critical role in which B cell specificities become activated and differentiate into long-lived B cell subsets. Notably, initial exposure to IAVs by vaccination will result in the preferential induction of B cells against epitopes of HA, as current influenza virus vaccines are enriched for HA from two IAVs (H1N1 and H3N2) and two influenza B viruses. As a result, B cells against multiple IAV HA subtypes included in the vaccine will be induced. However, multiple doses of inactivated vaccine are needed in immunologically naïve populations to reach protective levels of HAI^+^ antibodies [100,101], suggesting that inactivated vaccines poorly induce de novo B cell and T cell responses. In contrast, natural IAV infection recruits naïve B cells against all viral specificities, as the natural infective virus will express all viral antigens. However, if a particular subject’s first response to IAV is by infection, immunity will only be primed against the infecting IAV subtype. Therefore, first exposure to IAVs by different exposure routes primes the humoral immune response and long-lived B cell pools differently. As IAVs mutate frequently to escape host immunity, this long-lived humoral immunity generated by first exposure is limited to a given IAV strain and closely related strains. Consistent with this, children have a much narrower antibody-binding breadth than adults [102,103,104]. Notably, current adults were likely first exposed to IAVs during infancy and early childhood through natural infection with one IAV subtype. A person’s first exposure to a given IAV subtype increases with age, with nearly 100% of children seroconverting against at least one IAV subtype by the age of 6 in one study [105] and before the age of 2 in another study [106]. Seasonal influenza virus vaccinations in pediatric individuals provides an alternative imprinting route. Therefore, it is important to investigate how the exposure route, either infection or vaccination, ultimately affects future humoral immunity against drifted and novel IAVs.

### 3.2. Recall of MBCs and Naïve B Cells upon Repeated Exposures to IAVs

People tend to reuse MBCs that were generated during a childhood response to IAVs to respond to subsequent IAVs exposures, a phenomenon known as original antigenic sin [107], imprinting [108,109], and antigenic seniority [110]. Recalled MBCs target epitopes that are shared from prior exposures, which include both conserved and variable epitopes. Recalled MBCs can either rapidly differentiate into PBs or re-enter into GCs to further affinity mature against the inducing strain [4,98,111]. Notably, the MBC clones recalled into the GC and the PB pools are high affinity and are often clonally related [98,111,112], whereas low-affinity MBCs do not meaningfully participate in secondary responses or instead rapidly redifferentiate into MBCs [112]. In parallel, naïve B cells can be recruited into secondary GCs to generate a de novo B cell response, allowing for the diversification of the antibody response against shared and drifted viral epitopes [98,111]. As a result of secondary GCs, newly generated (naïve B cell-derived) or newly educated (MBC-derived) LLPCs and MBCs are generated to provide protection against the new viral variant.

Secondary GCs are comprised of both recalled MBCs and naïve B cells, with MBCs recalled into the GC demonstrating cross-reactivity with past strains and naïve B cells largely being strain specific [111]. Within secondary GCs, pre-existing MBCs and naïve B cells affinity mature, resulting in the generation of PBs and MBCs against these different specificities. Likely as a result of new naïve B cells being recruited against variable epitopes upon first exposure to pH1N1, secondary exposure to pH1N1 preferentially recalls MBCs targeting variable epitopes of the HA head [2,4,113]. Therefore, the hierarchy of recalled MBCs shifts towards variable epitopes of the HA head upon repeated exposure.

Which MBCs are recalled by viral exposure is highly dependent on the route of exposure, with vaccination largely recalling MBCs against HA [113,114] and infection largely recalling MBCs against viral antigens other than HA, including NA and NP [4,57]. Notably, MBCs that can cross-react with the new strain will be preferentially recalled, including MBCs with narrow and broad reactivity. Moreover, the epitopes that are preferentially targeted are dependent on the levels of cross-reactive antibodies that can bind the new strain and the antigenic distance between the strain that induced pre-existing MBCs and the new strain. For example, MBCs against conserved epitopes of the HA head and stalk domain were preferentially recalled in subjects first exposed to the pH1N1 virus in 2009, which was an antigenically novel strain and for which the population had low pre-existing immunity against [2,20,70,102,113,115]. Similarly, subjects that received experimental H5 and H7 vaccines preferentially recalled cross-reactive MBCs targeting conserved epitopes of the HA stalk [33,116,117,118,119,120]. As the antigenic distance between the new viral exposure and previously encountered viruses is large, subjects will preferentially recall broadly reactive MBCs, as was the case for first exposure to the pH1N1 virus and avian IAV subtypes. However, when people are exposed to the same or a similar IAV strain, people recall MBCs against variable epitopes of the HA head [2,4,20,113], as the antigenic distance between viruses is small.

### 3.3. Pros and Cons of Original Antigenic Sin

Despite its name, original antigenic sin is not necessarily always negative, as the recall of MBCs against protective epitopes can provide protection from IAV infection. Notably, the particular IAV subtype a person was first exposed to is often predictive of whether someone becomes infected and the severity of infection with an antigenically similar and distant strains or IAV subtype later in life [108,109,121]. Between 1918 and 1957, H1N1 viruses were the only circulating IAV subtype and therefore, most people born within this time frame were imprinted with an H1N1 virus that was antigenically similar to the 2009 pH1N1 virus [122]. During the 2009 pH1N1 outbreak, elderly people and older adults imprinted by historical H1N1 viruses in their youth were largely protected from severe disease and mortality [123] due to pre-existing immunity against conserved and variable epitopes of HA [20,124,125]. Subjects imprinted with a particular subtype are less susceptible to seasonal IAV infections with the same subtype later in life, with elderly adults more likely to be infected with H3N2 than H1N1 due to imprinting patterns [109,121]. Moreover, vaccination and infection with antigenically distant viruses can lead to the recall of MBCs with broad viral neutralization breadth, which could provide protection against antigenically distinct IAVs within the same HA group [126]. Noteworthily, the particular subtype a person was imprinted with as a child predicts whether subjects became infected or die due to infection with the avian IAV subtypes H5N1 and H7N9 [108]. Subjects that were initially infected with group 1 viruses (H1 and H2) during childhood were often protected from H5N1 infections, whereas subjects imprinted with a group 2 virus (H3) were more likely to be protected from H7N9 [108]. However, subjects imprinted with a virus of one HA group were more likely to become infected and die of an avian IAV from the other HA group [108]. Therefore, the recall of MBCs can boost antibodies against conserved protective epitopes within IAV groups. However, the preferential recall of MBCs against the imprinting IAV subtype may leave individuals susceptible to IAV infections with antigenically distant viruses, as subjects imprinted with a group 1 virus are more susceptible to infection with a group 2 virus and vice versa [108,109].

The preferential recall of cross-reactive MBCs from childhood exposures may also negatively impact humoral immunity against a drifted variant of the same subtype, as high-affinity neutralizing antibodies against a childhood strain may have a low affinity and may no longer neutralize the new strain [127]. Notably, antibodies isolated from subjects imprinted by H3N2 viruses in the 1960s and 1970s do not neutralize recent H3N2 viruses [128]. Despite this, the recall of MBCs may allow for the re-entry of low affinity B cells into GCs to affinity mature against the drifted viral variant. Notably, RBS-binding antibodies demonstrate strong features of original antigenic sin, as they often potently neutralize historical strains but not contemporary strains [12,19,20]. RBS-binding B cell clones can evolve over time, with B cells increasing affinity for new variants upon viral exposure, suggesting that these B cells can re-enter GCs and affinity mature against viral variants [19,20,129]. Therefore, the entry of MBCs into the GC allows for the fine-tuning of the antibody binding affinity for drifted viral epitopes. Moreover, the recruitment of naïve B cells into the MBC pool allows for the diversification of the anti-IAV B cell repertoire to new epitopes. Notably, vaccination has the potential to prophylactically re-train MBCs and recruit naïve B cells against new epitopes to provide potent protection against antigenically novel viruses, such as those observed during IAV pandemics.

Original antigenic sin is further exacerbated with age, as elderly people (>65 years old) tend to recall MBCs targeting conserved antigens [51,130]. Recalled MBCs often target conserved viral antigens, such as NP, but rarely induce antibodies against potently neutralizing epitopes of the HA head domain [51]. In accordance, older adults are less likely to seroconvert against influenza viruses and generally have lower neutralizing antibody titers than younger adults [131,132]. Moreover, MBCs from elderly subjects are less likely to re-enter into GCs and further affinity mature against drifted variants [51,133,134,135]. Notably, GC B cells in elderly mice and humans are noted for their reduced expression of activation-induced cytidine deaminase (AID) [136,137], an enzyme critical for somatic hypermutation. Additionally, elderly subjects have reduced Tfh cells following vaccination [138,139,140], which could contribute to the inability of B cells from elderly subjects to adapt to new IAV strains [51,130,135]. Moreover, hematopoiesis in elderly subjects is shifted towards the myeloid lineage, reducing the number of naïve B cells able to target drifted viral epitopes [141,142]. Therefore, mechanisms to overcome original antigenic sin are needed to produce protective antibody responses against circulating and novel IAVs within the elderly population.

## 4. Factors Limiting Robust Humoral Immunity and the Induction of bnAbs

For unclear reasons, MBCs against variable epitopes of the HA head are recalled upon repeated vaccination to IAVs, as opposed to MBCs targeting conserved protective epitopes of the HA head and stalk domains [2,22]. Upon IAV infection or in elderly subjects, people tend to recall MBCs against more conserved viral antigens, including NP and NA [4,38,51]. However, when exposed to a novel IAV, subjects tend to recall MBCs targeting conserved viral epitopes, including those found on the HA stalk [2,40,115,116]. The underlying mechanisms that dictate which B cell specificities are recalled by viral exposure, commonly called B cell immunodominance, are unclear. Below we discuss the factors that dictate which B cell specificities are recalled by IAV exposures.

### 4.1. Pre-Existing Serum Antibodies and Epitope Masking

As humans are regularly exposed to IAVs, people have pre-existing serum antibodies against a wide array of IAV antigens and epitopes. Pre-existing serum antibodies may limit the magnitude and specificity of the MBCs recalled by viral exposure. Notably, several studies have highlighted a negative correlation between pre-existing serum antibody titers and subsequent seroconversion upon vaccination [143,144,145], due to reduced MBC activation and PB responses [143,146]. As B cell activation is reduced by high pre-existing antibody titers, MBCs and naïve B cells are less likely to be recruited into GCs to undergo affinity maturation, limiting the development and maturation of humoral immunity against that IAV strain. Concordantly, de novo affinity maturation is blunted in subjects that are repeatedly vaccinated [147].

Pre-existing antibodies could reduce B cell activation after IAV exposure through multiple mechanisms, including rapid antigen clearance, immune complex formation, antibody feedback, and epitope masking. In the presence of pre-existing serum antibodies, viral antigens may be rapidly cleared, preventing the activation of MBCs and new naïve B cells. Moreover, pre-existing antibodies can form immune complexes with viral antigens that can regulate the few B cells that do become activated after IAV exposure. Follicular dendritic cells within the light zone of GCs acquire antigen via immune complexes, which can fine-tune GCs responses through competitive selection of clones into the LLPC and MBC pools [148]. To be positively selected, GC B cells must capture antigen from follicular dendritic cells to present to Tfh cells. Not only does a single GC B cell have to compete with other GC B cells, but GC B cells must also out-compete pre-existing antibodies within the immune complex for antigen acquisition, a process referred to as antibody feedback [149]. As a result, only the highest affinity GC B cells are able to acquire antigen and successfully compete for Tfh cells within the GC [149]. Therefore, an affinity bottleneck is created, with only a few high-affinity B cell clones being successfully selected, diminishing clonal diversity while increasing antibody affinity. However, complex antigens, such as HA, have been shown to drive asynchronous B cell selection, with B cells of varying affinities, including low and non-detectable affinities, selected to enter into the MBC population [150,151]. However, low-affinity MBCs or MBCs with undetectable affinities are less likely to participate in secondary GCs [98,112]. Moreover, recalled MBCs that comprise the PB response are dominated by high-affinity clones [4,99]. Therefore, the other overall role and utility of low-affinity MBCs clones remain unclear.

**Figure 3:**
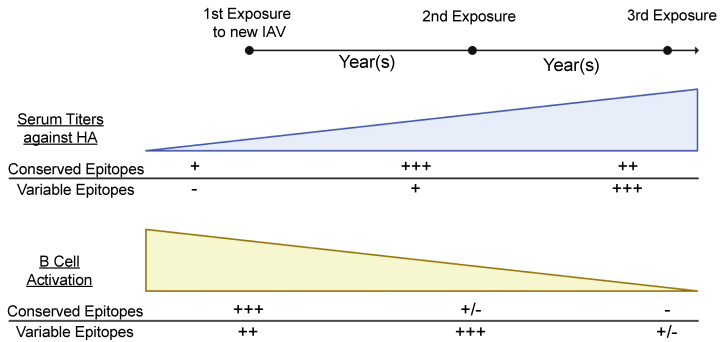
Serum titers and B cell activation following novel and repeated IAV exposure. People have low pre-existing serum titers against conserved epitopes shared with an antigenically distinct IAV. Upon first exposure, both naïve and memory B cells against the new IAV will induced and mature, increasing serum antibody titers against both conserved and variable epitopes. Upon second exposure, only B cells against variable epitopes will be recalled, with serum antibodies increasing against corresponding epitopes. Upon third exposure, serum titers are high against all epitopes and B cells will not become activated against any epitopes.

The particular epitope-specific MBC clones that are recalled upon IAV exposure may, in part, be dictated by the levels of pre-existing serum antibodies that can bind a particular epitope, a phenomenon known as epitope masking (Figure 3). Antibodies binding a specific epitope can limit the induction of more antibodies against the same epitope, but not other epitopes [152,153,154]. Therefore, epitope masking may lead to the diversification of the humoral immune response against multiple epitopes, rather than continually recalling MBCs against the same epitopes. If the antigenic distance between the viruses the subject has previously been exposed to and the current IAV exposure is large, that subject will have low pre-existing serum antibodies against most epitopes of HA, including the HA stalk domain, and may not possess MBCs against variable epitopes of the HA head (Figure 3). In this scenario, subjects can recall MBCs targeting conserved protective epitopes of the HA head and stalk domains and recruit naïve B cells against variable epitopes of the HA head (Figure 3) [2,115,116,117,120,155]. Afterwards, subjects will likely have moderate titers against conserved HA epitopes but still relatively low levels of antibodies against the variable antigenic sites. Upon secondary exposure to the same or a similar IAV, MBCs against the variable epitopes can be recalled by secondary exposure, whereas conserved epitopes are masked by pre-existing antibodies, limiting MBC recall against these epitopes. As pre-existing antibody titers are now high against all epitopes, MBC recall will be significantly blunted upon subsequent exposures to the same or a similar virus (Figure 3), leading to the reduced PB response phenotype observed in repeated vaccination cohorts [143,144,145].

It is not known what level of antibodies is necessary to limit B cell activation against particular epitopes. Many people have low pre-existing serum antibodies against the HA stalk domain that could suppress the recall of MBCs against stalk domain epitopes [111,156,157]. Notably, one study of anti-*Plasmodium* antibodies identified that non-protective levels of pre-existing antibodies were sufficient to limit B cell activation [152]. Thus, non-protective levels of anti-HA antibodies could limit B cell activation, further affinity maturation against drifted variants, and protective antibody responses against IAVs.

### 4.2. Competition for Antigen, T Cell Help, and Niches

The particular B cell specificities that are selected into the long-lived humoral immune pool are dependent on the ability of a particular B cell clone to outcompete other B cell clones for antigen. B cell clones targeting the same antigen, such as HA, but distinct epitopes, compete for the same antigen within the GC, with the highest affinity B cell clones being preferentially selected in the LLPC pools. In addition, MBCs with appreciable affinity are more likely to undergo further affinity maturation in secondary GCs, whereas low-affinity MBCs do not re-enter into secondary GCs and can rapidly redifferentiate into MBCs [112]. Notably, strain-specific PBs are typically higher affinity for the inducing IAV strain, whereas PBs against conserved epitopes are typically higher affinity for past strains [4]. As a result, recalled MBCs against conserved epitopes are likely at an affinity disadvantage in GCs relative to strain-specific MBCs in the response to new IAVs.

Conserved epitopes of HA are often difficult to access, limiting the ability of B cells to bind these epitopes and become activated. B cells against the HA stalk may be disadvantaged in terms of their ability to reach these epitopes because the HA stalk domain sits within the viral membrane and virions are highly decorated with glycoproteins, limiting access to these epitopes [158,159]. Notably, HA stalk-binding antibodies have reduced affinity for the full virus relative to recombinant HA, as the viral membrane and nearby glycoproteins reduce accessibility [2,46]. Moreover, the RBS on the HA head is an exceptionally small epitope, with most antibodies using only heavy chain complementarity-determining region 3 (CDR3) to bind the conserved residues of the RBS [12,14]. As a result of reduced accessibility, B cells targeting these epitopes likely have lower avidity, or a combination of the affinities of multiple antibody-binding sites. Avidity functions in several ways: the avidity of the two binding sites of a single antibody molecule, or BCR on the surface of a B cell, and the combined avidity of multiple BCRs on a single B cell. If an epitope is difficult to access, it is unlikely that both binding sites of a BCR will be able to bind antigen or that multiple BCRs on a B cell will be able bind antigen, a process known as BCR clustering. In the absence of BCR clustering, B cells are less efficient at internalizing antigen that can be presented to CD4 T cells [160,161]. Notably, B cells with higher avidity are more likely to be immunodominant [162]. Thus, B cells targeting difficult-to-reach epitopes will be less likely to become activated and differentiate into PBs or enter GCs.

B cells also have to compete for CD4 T cell help to become activated and selected in GCs. The highest affinity clones are preferentially activated, as they will be more likely to take up antigen and present it to CD4 T cells. Additionally, the particular antigen-specific clones that are activated is dependent on the number of B cells targeting the same antigen as antigen-specific CD4 T cells. Notably, IAV-specific CD4 T cells largely target NP and M1, rather than HA and NA [163], although circulating Tfh cells largely target HA [164]. However, it is not known what the specificities of activated CD4 T cells are within the draining lymph nodes, the locations of B:T cell interactions. Moreover, B cell immunodominance is in part dependent on early CD4 T cell help [165], indicating competition for CD4 T cell help is important for determining B cell immunodominance. Further research is required to understand how CD4 T cell specificity shapes the activation and selection of antigen-specific B cells in the context of influenza viruses.

Antibody responses against IAVs decline over time for unclear reasons. Notably, a recent study identified that vaccine-induced LLPCs within the bone marrow decline 1 year following influenza virus vaccinations in adults [166]. This study suggests that either LLPCs do not acquire the proper transcriptional and metabolic profile required for LLPC differentiation or maintenance, or that the newly formed LLPCs cannot compete for the limited LLPC niches available in the bone marrow. As humans are exposed to IAVs throughout their lifetime, it is unlikely that all LLPCs that bud off GCs can successfully compete for space within the bone marrow. Moreover, antibody feedback could limit LLPC survival, as antibody-mediated FcγRIIβ crosslinking on LLPCs can induce apoptosis [167]. Therefore, newly generated LLPCs may not successfully compete for space within the limited niches required for LLPC persistence. However, the precise mechanisms that limit LLPC generation and maintenance remain unclear.

### 4.3. Restricted B Cell Repertoires and Polyreactivity/Autoreactivity of bnAbs

B cells targeting conserved epitopes frequently use BCR repertoires with restrictive features, including specific V(D)J gene usages and binding motifs. Notably, most antibodies targeting the broadly neutralizing stalk epitope of group 1 IAVs utilize a single VH gene, VH1-69 [168,169]. VH1-69-expressing B cells often express a patch of hydrophobic residues within the H-CDR2 loop that are critical for antigen binding. Additional classes of stalk-binding antibodies and antibodies targeting the lateral patch of the HA head similarly utilize restricted repertoire features that are critical for epitope binding [20,42,43,46]. Moreover, RBS-binding antibodies typically possess long H-CDR3s, which mimics sialic acid binding [12,14,15]. Thus, the number of B cells specific to conserved epitopes of the HA head and stalk domains are confined by the number of B cells with these restrictive repertoire features.

Antibodies targeting conserved epitopes of HA are often polyreactive [2,113], the ability of a single antibody to bind to multiple unrelated molecularly distinct antigens. The polyreactivity of bnAbs is associated with increased viral binding breadth, affinity, and neutralization potential against a wide array of IAVs [113], indicating it may function as a stopgap to provide protection against antigenically distinct IAVs. However, polyreactive antibodies can bind self-antigens [113,170,171] and, as a result, may be negatively selected to avoid self-reactivity. Moreover, polyreactivity is inherent to naïve B cells that target conserved epitopes, which are preferentially selected into the MBC pool against broadly neutralizing epitopes [113]. As polyreactivity and autoreactivity are major contributors to clonal deletion [172], the repertoire of naïve B cells against conserved epitopes of HA may be further reduced. Moreover, many anergic, or hyporesponsive, naïve B cells are polyreactive [173] and atypical MBCs induced by *Plasmodium* infection are commonly polyreactive [174]. Therefore, a fine balance between allowable self-reactivity and increased adaptability to novel IAVs is required for optimal bnAb responses. Further studies on the role of polyreactivity in limiting bnAb responses are required to understand how to safely overcome these immunological barriers in order to elicit polyreactive bnAbs.

## 5. Vaccine Strategies to Overcome B Cell Immunodominance

New vaccine strategies are needed to provide broad and long-lived protection against IAVs. Here, we discuss new vaccine strategies to preferentially induce antibodies against conserved epitopes of HA and mechanisms to overcome pre-existing serum antibodies against both conserved and variable epitopes of HA.

### 5.1. Removing, Replacing, or Masking Variable Epitopes of HA

The major limitation of current influenza virus vaccines is that they tend to recall MBCs against variable epitopes of the HA. Several next-generation influenza virus vaccines are designed to preferentially recall MBCs against conserved epitopes of HA while avoiding responses against variable epitopes. The mini-HA and headless HA constructs possess only the stalk domain, with the head domain completely removed [175,176]. Use of the mini-HA/headless HA immunogens in non-human primates showed the robust induction of protective antibodies against the HA stalk, suggesting that this vaccine strategy could similarly induce stalk antibodies in humans [177,178]. Similar in concept, the chimeric HA (cHA) strategy maintains the stalk domain from seasonal H1 or H3 viruses but replaces the head domain with a head domain from an avian IAV subtype [179,180]. As people have little to no pre-existing immunity against the variable epitopes of avian IAV subtypes, people would preferentially recall MBCs against conserved epitopes on the HA stalk. Notably, a phase I clinical trial of an H1 cHA vaccine trial showed humans could recall MBCs against multiple conserved epitopes of the HA stalk domain and the trimer interface epitope of the HA head [46,157,181].

However, focusing the antibody response against only a few epitopes on the HA stalk could lead to natural escape mutations, which could make these vaccines obsolete. Therefore, vaccine strategies that induce antibodies against conserved protective epitopes of both the HA head and stalk are critical for durable protection against IAVs. To that end, mosaic HA immunogens replace only the variable epitopes of the HA head with the variable epitopes of an avian HA subtype [7,182] and have been shown to induce broadly reactive antibodies against both the HA head and stalk domains [182]. Moreover, masking the variable epitopes of the HA head with glycans [24,183] can similarly divert the antibody responses towards conserved epitopes of the HA head and stalk domains. Lastly, computationally designed antigens that use the most conserved HA sequences similarly can induce antibodies against conserved epitopes of HA [184,185,186]. Therefore, multiple strategies that remove or obscure the variable epitopes of HA are showing promise in terms of their recruitment of B cells against multiple conserved epitopes of HA.

### 5.2. Vaccine Formulations to Increase B Cell Activation and bnAb Induction

One hurdle to inducing protective levels of bnAbs is overcoming pre-existing serum titers and sufficiently cross-linking BCRs on MBCs. Notably, the subjects enrolled in the cHA vaccine clinical trial were refractory to further inducing serum antibody titers against the HA stalk in response to the boosted dose, despite receiving a second heterologous (different head, same stalk) cHA construct [157,181]. Therefore, vaccines should be formulated to overcome the issues of pre-existing immunity and the limited avidity of B cells targeting conserved epitopes of HA.

Several vaccine platforms and formulations that are intended to overcome pre-existing serum antibodies are in various stages of clinical development and licensure. High-dose HA vaccines are licensed for use in humans ≥ 65 years old to overcome the issues of pre-existing serum antibodies and immune senescence. High-dose vaccines possess 4× more HA antigen than standard vaccines and have shown an increased capability of driving seroconversion and protection from influenza-like disease [187]. By increasing the amount of antigen, pre-existing antibodies will not be able to sequester all antigen present within the vaccine, allowing for free antigen to facilitate MBC activation and thus promote seroconversion against protective epitopes of HA. However, the high-dose vaccines still drive the antibody responses against the HA head, limiting the potential antibody breadth [187].

Oil-in-water adjuvants have been shown to dramatically increase cross-reactive antibody responses following vaccination relative to non-adjuvanted vaccines [177,188,189]. Oil-in-water adjuvants function to emulsify viral antigens within the adjuvant, which may protect antigen from being bound by pre-existing serum antibodies and may allow for the draining of unprocessed antigen to lymph nodes, as well as the stimulation of innate immune pathways [190,191]. Influenza virus vaccines that use oil-in-water adjuvants are noted for their ability to activate and promote the affinity maturation of both naïve B cells and MBCs [192,193]. Furthermore, a phase I clinical trial of the group 1 cHA vaccine revealed that only subjects that received the inactivated vaccine with the adjuvant AS03 substantially seroconverted against the HA stalk domain [46,157,181]. Taken together, these studies indicate the critical role of adjuvants in promoting antibody responses against conserved epitopes of HA.

Numerous studies have shown that membrane-bound antigen is more immunogenic than soluble antigen [194,195], likely by driving BCR clustering and enhanced BCR signaling. As many B cells targeting broadly neutralizing epitopes are polyreactive and potentially anergic [113], these B cells may require stronger BCR signaling to become activated. Therefore, vaccine strategies that cluster HA on a membrane may ideally stimulate humoral immunity. One strategy to increase BCR crosslinking uses nanoparticles that display diverse HA antigens in which adjacent HAs are heterotypic and antigenically distinct [196,197]. The use of the heterotypic nanoparticles limits the ability of strain-specific B cells to crosslink multiple BCRs across multiple HAs, while conferring an advantage to B cells targeting conserved epitopes that are able to crosslink multiple BCRs across adjacent heterotypic HAs. Additionally, headless HA nanoparticles likely function in a similar manner to expose the conserved epitopes of the HA stalk domain, allowing for BCR crosslinking across headless HA units and promote B cell responses against the HA stalk [177,198]. Moreover, mRNA vaccines or adenovirus-vectored vaccines that express membrane-bound antigen have the potential to provide broad and potent humoral immunity against IAVs [199,200].

Vaccines should also be optimized to promote the selection of B cells targeting conserved epitopes within the GC. One such strategy is the use of iterative vaccine strategies, where sequential immunizations continually deliver antigen to GCs. As B cells targeting conserved epitopes may be at an affinity disadvantage, increasing the amount of available antigen by sequential immunizations can allow for permissive B cell selection by reducing affinity-based competition for antigen. Notably, sequential immunizations with HA in pre-clinical models has been shown to increase bnAb responses [201,202]. Alternatively, one dose of a vaccine that slowly delivers antigen over time, as in the case of adenovirus-vectored vaccines [203] and microneedle patch vaccinations [204,205], could drive the maturation of GC B cells expressing bnAbs. Taken together, novel vaccination platforms and formulations have the potential to induce broadly neutralizing antibodies against influenza viruses.

## 6. Conclusions

Humoral immunity against IAVs is limited by the inability of the host immune system to induce and maintain antibody responses against conserved protective epitopes. Neutralizing antibodies against conserved epitopes of HA have the greatest potential to provide robust and broad protection against IAVs but are rarely induced. Multiple factors limit the induction of bnAbs. B cells targeting conserved epitopes frequently use restricted repertoire features and are often polyreactive, suggesting that the naïve B cell repertoire against these epitopes is limited. MBCs targeting conserved epitopes must compete with strain-specific B cells for antigen. As MBCs against conserved epitopes tend to have a stronger affinity for past strains, they are likely to be outcompeted by strain-specific MBCs that have a high affinity for current IAV antigens. Moreover, pre-existing serum antibodies can lead to the rapid clearance of antigen and mask-conserved epitopes, limiting the activation and further affinity maturation of MBCs against conserved epitopes. Similarly, antibody feedback can limit GC responses and limit bnAb responses. Lastly, B cells targeting conserved epitopes are at an avidity disadvantage, as many conserved epitopes are difficult to reach.

New vaccine formulations and platforms are being developed to circumvent these issues. Notably, a phase I clinical trial with a cHA showed the robust recall of MBCs against multiple conserved epitopes of the stalk domain. Pre-clinical vaccines that overcome issues of B cell avidity, including those that use nanoparticles, have also been shown to induce antibodies against conserved epitopes of the HA head and stalk domains. Further research is required to understand how vaccination can overcome the limitations outlined in this review, including overcoming and preventing viral evolution around broadly neutralizing antibodies and how to properly harness MBCs against conserved epitopes to provide durable humoral immunity against IAVs.
